# The Effect of Arginine Supplementation on Intestinal Antioxidant Capacity, Whole Blood Cell Count and Antiviral Immune Function of Piglets Infected with Porcine Epidemic Diarrhea Virus

**DOI:** 10.3390/ani16132002

**Published:** 2026-06-30

**Authors:** Zhiwei Zhang, Yunlong Du, Rongrong Jian, Hanbo Li, Zhonghua Li, Peng Li, Lei Wang, Di Zhao, Dan Yi, Tao Wu, Mengjun Wu, Yongqing Hou

**Affiliations:** 1 Hubei Key Laboratory of Animal Nutrition and Feed Science, Wuhan Polytechnic University, Wuhan 430023, China; 18240546296@163.com (Z.Z.); 19042608715@163.com (Y.D.); 15225319692@163.com (R.J.); lhb987750857@163.com (H.L.); yuxincangqiong@163.com (Z.L.); lp1536698031@163.com (P.L.); wanglei_wh@aliyun.com (L.W.); zhaodi24@163.com (D.Z.); yidan810204@163.com (D.Y.); wtao05@163.com (T.W.); 2 Engineering Research Center of Feed Protein Resources of Agricultural By-Products, Ministry of Education, Wuhan Polytechnic University, Wuhan 430023, China

**Keywords:** arginine, weanling piglets, intestinal damage, porcine epidemic diarrhea virus

## Abstract

Porcine epidemic diarrhea virus (PEDV) imposes substantial economic losses on the global swine industry owing to its high pathogenicity and transmissibility. Although arginine (Arg) is known to support the integrity of intestinal barrier, it is not clear whether Arg can alleviate intestinal injury induced by PEDV. A total of 32 healthy 7-day-old piglets were randomly assigned to four groups (Control, Arg, PEDV, PEDV + Arg; eight replicates per group). From day 5, piglets in the Arg and PEDV + Arg groups were orally administered Arg at 400 mg/kg body weight until day 11; then, PEDV (1 × 10^5.5^ TCID_50_) was given orally for two PEDV-infected groups. On day 14, all piglets were slaughtered to obtain blood and intestine samples for further analysis. The results showed that PEDV infection significantly reduced T-SOD and CAT activities in plasma and intestine while elevating MPO levels. Arg supplementation restored T-SOD (plasma, duodenum, ileum), CAT (plasma, ileum), and GSH-Px (jejunum, ileum) activities and reduced MDA (jejunum) content in PEDV-infected piglets. Hematological analysis showed Arg alleviated PEDV-induced increases in MCV and RDW-SD, and significantly elevated MCHC. The real-time quantitative PCR analysis demonstrated that Arg further enhanced PEDV structural genes (*M*, *N*, *S*) expression in the duodenum, ileum, and colon. Concurrently, Arg significantly up-regulated interferon-stimulated genes (*MX1, OASL, ISG15, IFITM3*) in the ileum, *IRF7* in the duodenum and colon, and *IFN-β* in the ileum. Arg also down-regulated the pro-inflammatory cytokines *IL-6* and *CXCL2* and the antimicrobial peptide *REG3G* in the colon, while up-regulating the tissue repair gene *MMP13* in the ileum. In conclusion, oral Arg exhibits a unique dual role: it promotes PEDV replication to a certain extent while significantly enhancing antioxidant capacity, strengthening intestinal antiviral immunity, and attenuating intestinal inflammation. These findings highlight Arg’s role in promoting disease tolerance and offer a novel perspective for nutritional intervention strategies against PEDV infection.

## Simple Summary

Porcine epidemic diarrhea virus causes severe intestinal damage in piglets, leading to high mortality and major economic losses in pig farming. Arginine is an amino acid known to support gut health, but whether it can protect against PEDV-induced intestinal injury remains unclear. In this study, we gave arginine to PEDV-infected piglets and examined their blood and intestinal tissues. We found that arginine improved the body’s antioxidant defenses, strengthened intestinal antiviral immune responses, and reduced inflammation. Interestingly, arginine also appeared to slightly promote viral gene expression, suggesting a unique disease-tolerance mechanism, allowing the host to better cope with viral infection rather than simply fighting it. These findings offer a new nutritional strategy for managing PEDV infection in piglets and highlight arginine’s potential as a functional feed additive for improving gut resilience during viral challenges.

## 1. Introduction

Porcine epidemic diarrhea virus (PEDV) is a highly contagious enteric pathogen that causes acute diarrhea, vomiting, dehydration, and high mortality in neonatal piglets, leading to huge economic losses in the global swine industry [1,2,3]. PEDV belongs to the genus *Alphacoronavirus* within the family *Coronaviridae* and the order *Nidovirales*. It is an enveloped, single-stranded positive-sense RNA virus that encodes three major structural proteins: spike (S), membrane (M), and nucleocapsid (N) [4,5]. The S protein plays a critical role in viral entry by binding to host-cell receptors and mediating membrane fusion [6]. In recent years, due to the diversity of *S* genes, highly pathogenic PEDV mutants appeared and spread rapidly around the world, which led to a significant decline in the protective effect of traditional vaccines [5,7,8,9].

L-Arginine (Arg) is a key metabolic substrate under physiological or environmental stress. Arg is not an essential amino acid, but it becomes an essential amino acid under certain conditions [10]. It is not only the basis of protein synthesis, but also the only precursor of nitric oxide (NO) synthesis in the body [11]. Arg can regulate intestinal blood flow, promote vascular growth and intestinal stem cell renewal, and alleviate intestinal injury through mTOR signaling pathway [12,13,14]. Studies have shown that oral administration of Arg can improve the intestinal villus morphology of PEDV-infected piglets and up-regulate the expression of tight junction proteins [15]. In addition, other studies have shown that arginine can enhance the activities of glutathione peroxidase and superoxide dismutase in plasma and alleviate the oxidative stress of weanling piglets induced by diquat. At the same time, it can alleviate the overexpression of *IL-6* induced by lipopolysaccharide (LPS) [16,17]. Dou et al.’s research also showed that arginine could enhance the activity of antioxidant enzymes (CAT, GSH-Px) and decrease the relative expression of intestinal inflammatory factor *IL-6* [18]. However, there are few studies on its alleviation of intestinal damage in piglets infected with PEDV.

The present study was designed to investigate the effects of oral arginine supplementation on intestinal antioxidant function and the immune barrier of piglets infected with PEDV, and provide a theoretical basis for developing functional feed additives and improving the economic benefits of the global pig industry. In addition, the present study also sought to understand how Arg modulates host tolerance to PEDV infection, a critical aspect often overlooked in nutritional interventions.

## 2. Materials and Methods

### 2.1. Animal Experiment

The animal experiment was completed in the animal room of the biosafety laboratory of the Institute of Animal Husbandry and Veterinary Medicine, Jiangxia District Academy of Agricultural Sciences, Wuhan City, China. The animal room was thoroughly cleaned and fumigated (potassium permanganate + formaldehyde) before the animal experiment. During the experiment, the ventilation of the enclosure was well maintained, and the temperature in the enclosure was stable at 27–29 °C. In this experiment, 32 7-day-old healthy three-way hybrid piglets (Duroc * Large White * Landrace) of the same source and similar body weight were selected. Thirty-two piglets were randomly divided into four treatment groups with control group, Arg group, PEDV group and PEDV + Arg replicates in each group, and one pig in each replicate. Each treatment group was distributed in an independent and closed enclosure, and the piglet house maintained the same conditions and strictly controlled the cross-infection of the piglet house. The experimental diet was imported full-fat milk powder from Nouriz’s New Zealand and purchased from Nouriz’s Company (Shanghai City, China). The nutritional components are detailed in Table A1. During the experiment, each group was fed the same basal diet (Nouriz whole milk powder was mixed with warm water at 45–55 °C according to the ratio of milk powder to water of 1:5, free access to food and water). The experiment period was 14 days: the first 4 days was the adaptation period, and days 5–11 constituted the treatment period. Arginine (400 mg/kg BW) was orally given to the Arg group and the PEDV + Arg group, and the other groups were given the same volume of whole milk powder. On the 11th day of the experiment, PEDV (1 × 10^5.5^ TCID_50_) was orally given to the PEDV group and the PEDV + Arg group, and the other groups were orally given the same volume of PBS solution.

### 2.2. Sample Collection

On the 13th night (21:30), all piglets were stopped from supplying water and feeding. On the 14th morning (6:30), blood was collected from the piglet’s anterior vena cava with an EDTA anticoagulant vacuum blood collection tube (KWS, Shijiazhuang, China) and No.7 blood collection needle (Aosaite, Heze, China). Three tubes of 3.5 mL blood samples were centrifuged at 3500 r/min for 15 min, and the supernatant was taken to prepare plasma. Whole blood and plasma were divided into 1.5 mL centrifuge tubes (Labselect, Beijing, China) and stored in a refrigerator at −80 °C. Following blood collection, all piglets were euthanized by intravenous administration of sodium pentobarbital (50 mg/kg BW, Zoletil, Shanghai, China). Immediately after confirmation of death, the abdominal cavity was opened, and intestinal segments were harvested. Contents were gently flushed with physiological saline before tissue collection. Intestinal tissue was cut into small pieces at low temperature, wrapped in tin foil, quickly frozen in liquid nitrogen, and transferred to a refrigerator at −80 °C to store samples.

### 2.3. Whole Blood Cell Count Analysis

A Siemens ADVIA ^®^ 2120i automatic blood cell analyzer (Siemens, Munich, Germany) was used to test white blood cell count (WBC), neutrophil count (Neu), lymphocyte count (Lym), eosinophil count (Eos), basophil count (Bas), platelet count (PLT), red blood cell distribution width standard deviation (RDW-SD), red blood cell distribution width coefficient of variation (RDW-CV), mean platelet volume (MPV), red blood cell count (RBC), hemoglobin (HGB), mean red blood cell hemoglobin concentration (MCHC), mean corpuscular hemoglobin content (MHC), platelet distribution width (PDW) and other indicators.

### 2.4. Antioxidant Capacity and Oxidation-Relevant Products in the Plasma and Intestine

Plasma, jejunum, ileum, colon and duodenum samples were used for the analysis of antioxidant enzymes and related products. Catalase (CAT), hydrogen peroxide (H_2_O_2_), total superoxide dismutase (T-SOD), malondialdehyde (MDA), myeloperoxidase (MPO) and glutathione peroxidase (GSH-Px) were measured. All the above indicators were measured by the kit purchased by Nanjing Jiancheng Bioengineering Institute (Nanjing, China), and all the steps were strictly manipulated according to the kit instructions.

### 2.5. RNA Isolation and Quantitative Real-Time PCR

The relative expression levels of genes in duodenum, ileum and colon were measured. The related detection gene sequences are shown in Table A2. Total RNA was extracted from intestinal tissue samples by TRIzol (Takara, Dalian, China) method. The concentration and purity of the extracted RNA were analyzed by a Thermo Fisher Scientific NanoDrop 2000 (Shanghai, China) ultramicro spectrophotometer according to the instructions of the RNAiso Plus kit. Reverse transcription is performed according to the method steps of the PrimeScript RT reagent kit with gDNA Eraser kit (Takara, Dalian, China). Subsequently, real-time fluorescence quantitative PCR was performed using SYBR Premix Ex Taq (Takara, Dalian, China). Ribosomal protein L19 (*RPL19*) was used as an internal reference for the PCR results. According to the method of Fu et al. [19], the relative expression level of the gene was calculated by 2^−ΔΔCt^ calculation relative expression level algorithm, and statistical analysis was performed. The genes analyzed included PEDV structural genes (*M, N* and *S*), antiviral genes (*MX1, ISG15, IFITM3, OASL, IRF7*, *IFN-β*), intestinal inflammatory cytokines (*IL6, IL8, CXCL2, IL-1β, REG3G*) and tissue repair genes (*AREG, MMP13*).

### 2.6. Statistical Analysis

The experimental data were analyzed by SPSS 21.0 software (IBM, Chicago, USA). A general linear model (multivariate ANOVA) was applied to examine the effects of PEDV infection status (non-infection and infection), Arg supplementation (addition and non-addition), and their interaction on plasma and intestinal antioxidant indices, gene expression levels in duodenum, colon and ileum, and hematological parameters. Multiple comparisons among groups were performed using Tukey’s honest significant difference (HSD) test. Differences were considered statistically significant at *p* < 0.05. Data are presented as mean values ± standard error of the mean (SEM). If different columns in the table do not share the same lowercase letter, the difference is significant (*p* < 0.05).

## 3. Results

### 3.1. Effects of Arginine Supplementation on Antioxidant Status in PEDV-Infected Piglets

Compared with the control group, PEDV infection decreased the levels of catalase (CAT) in plasma, duodenum and jejunum (*p* < 0.05), as well as total superoxide dismutase (T-SOD) levels in plasma and ileum, and increased the content of myeloperoxidase (MPO) in colon (*p* < 0.05); compared with the PEDV group, after adding Arg, the activities of antioxidant enzymes and related products in the intestines and plasma of piglets were significantly changed, the contents of CAT and T-SOD in plasma were significantly increased (*p* < 0.05), and the T-SOD in duodenum was significantly increased (*p* < 0.05); the glutathione peroxidase (GSH-Px) in jejunum was significantly increased and the malondialdehyde (MDA) content was significantly decreased (*p* < 0.05), and the contents of T-SOD, CAT and GSH-Px in ileum were significantly increased (*p* < 0.05) (Table 1).

### 3.2. Effect of Arginine Supplementation on Complete Blood Count in Piglets Infected with PEDV

Compared with the control group, the mean corpuscular volume (MCV) and red blood cell distribution width (RDW-SD) of the PEDV group showed an upward trend, and the mean corpuscular hemoglobin concentration (MCHC) showed a downward trend; compared with the PEDV group, after adding Arg, the upward trend of MCV and RDW-SD was alleviated, and MCHC was significantly increased (*p* < 0.05) (Table 2).

### 3.3. Effect of Arginine Addition on Expression of Intestinal Related Genes in Piglets Infected by PEDV

Compared with the control group, the viral structural genes *M*, *N* and *S* in the duodenum, ileum and colon of the PEDV infection group were significantly increased, and the expression was further enhanced after administration of arginine (*p* < 0.05) (Table 3). At the same time, PEDV infection significantly increased the expression of antiviral genes (*MX1*, *OASL*, *ISG15*, *IFITM3*) in duodenum and ileum, and was further improved after arginine administration (*p* < 0.05) (Table 4). Compared with the PEDV infection group, Arg could significantly down-regulate the expression of *IL-6, CXCL2* and *REG3G* genes in colon (*p* < 0.05) (Table 5), and significantly up-regulated ileum tissue repair gene (*MMP13*) (*p* < 0.05) (Table 4).

## 4. Discussion

Porcine epidemic diarrhea virus (PEDV) is a coronavirus that induces acute intestinal damage, villus atrophy and poor absorption in newborn piglets, which brings huge economic losses to the global pig industry [1,2,20]. The destruction of intestinal epithelial barrier and the induction of a severe inflammatory reaction are the main characteristics of PEDV [7,8]. Nutritional interventions, especially the supplementation of functional amino acids, have received extensive attention due to their potential to regulate host immune responses and maintain intestinal health during viral challenges [21,22]. Arg is a conditionally essential amino acid that plays a key role in maintaining intestinal mucosal integrity, regulating immune response and alleviating oxidative stress in weanling piglets [23,24]. In this study, the effects of oral arginine on intestinal gene expression, antioxidant function and the whole blood cell count of piglets infected with PEDV were determined, and the positive effect of arginine on alleviating intestinal injury in weanling piglets infected with PEDV was explored.

Oxidative stress is a key factor in the pathogenesis of PEDV infection, which usually leads to the accumulation of reactive oxygen species (ROS) and subsequent damage to the intestinal epithelium [25,26]. Previous studies have shown that PEDV infection significantly reduced the activity of superoxide dismutase (SOD) and catalase (CAT) in the intestine of piglets, and increased the level of lipid peroxidation products (MDA) [27,28,29]. The results of this experiment showed that PEDV infection reduced the levels of T-SOD and CAT in plasma and small intestine, and increased the content of MPO in colon, which was consistent with the previous experimental results. After oral administration of 400 mg/kg BW Arg, the activities of T-SOD, CAT and GSH-Px in the intestinal tract of PEDV-infected piglets were significantly increased, and the level of MDA in the jejunum was decreased. This result is consistent with the research results of Zheng et al. in inducing the oxidative stress model of weanling piglets in diquat. Adding 1.0% Arg to the diet increased the activities of SOD and GSH-Px in the jejunum of diquat infected piglets, and decreased the content of MDA [22]. Other studies have shown that arginine can enhance the antioxidant defense system and reduce oxidative stress induced by LPS in weanling piglets [16,17]. In addition, arginine is a multifunctional metabolic substrate and a precursor for the synthesis of protein, nitric oxide (NO), polyamines and creatine, which are essential for cell redox balance and mucosal repair [11,12]. Therefore, the addition of 400 mg/kg BW Arg enhanced the antioxidant capacity of the body, thereby protecting the intestinal tract of piglets from oxidative damage caused by PEDV infection.

Whole blood cell count provides a meaningful reference for understanding the systemic effects of viral infection. For example, PEDV can exploit red blood cells and spread to the whole body, resulting in intestinal infection damage [30]. The results of this experiment showed that PEDV infection caused an increase in MCV and RDW-SD and a decrease in MCHC. It shows that PEDV infection leads to larger red blood cells and increased heterogeneity of red blood cell size, which is consistent with the oxidative damage of red blood cell membrane [29,31]. Oral administration of 400 mg/kg BW Arg alleviated the upward trend of MCV and RDW-SD, and significantly increased MCHC. Oxidative stress caused by PEDV infection damaged erythrocyte membrane, which led to the increase in MCV, the increase in RDW-SD and the decrease in MCHC. After adding Arg, the antioxidant function of the body was enhanced, the antioxidant enzyme activity in intestine and plasma was restored, and the content of oxidation products was reduced, which alleviated the damage of erythrocyte membrane caused by oxidative stress, thus alleviating the rising trend of MCV and RDW-SD. According to Masiuk et al.’s [32] research, piglets infected with PEDV at the age of 1–7 days have increased red blood cells, white blood cells, monocytes, hemoglobin and hematocrit 3–5 days after clinical symptoms appear. In addition, the research of De Arriba et al. [33] showed that the strong lymphocyte proliferation reaction in peripheral blood could be detected on the 4th–21st day after inoculation with virulent PEDV, and the response induced by attenuated strain was weak. In contrast, in this experiment, blood samples were collected on the morning of the third day after PEDV inoculation, which was not only earlier than the observation window of blood concentration (3–5 days) confirmed by Masiuk et al. [32], but also earlier than the initial time of the lymphocyte proliferation reaction (4 days) reported by De Arriba et al. In addition, the PEDV strain used in this experiment was YN strain, which was different from the CV-777 strain used by De Arriba.

In order to further understand the role of Arg in piglets infected with PEDV, the viral structural genes, antiviral genes, intestinal inflammatory cytokines and tissue repair genes in duodenum, ileum and colon were detected in this experiment. The results showed that PEDV infection significantly up-regulated the viral structural genes, which was consistent with the results of PEDV infection intestinal injury models [15,34,35]. After oral administration of Arg, the expression of viral structural genes was further enhanced. This result is consistent with the study of Shi et al. [15], who indicated that Arg can enhance the expression of viral structural genes, but has a protective effect on the whole intestine. This suggests that Arg may have two effects, in maintaining the intestinal barrier while also facilitating transcription of viral genes. Arg is a multifunctional metabolic substrate and a precursor for the synthesis of protein and polyamines, which can promote virus proliferation under certain conditions [36,37]. Previous studies have found that African swine fever virus and some coronaviruses can hijack amino acid metabolism, and then facilitate viral transcription [38]. In addition, according to Shi et al.’s [15] multi-omics study, it was found that the promotion of Arg on PEDV replication may be due to the high arginine content in viral proteome. Arginine is one of the most abundant amino acids in *PEDV N* protein, which can facilitate transcription of PEDV by enriching the synthetic substrate of N protein [28,36,39]. In addition, excessive or prolonged arginine supply may destroy the balance between metabolism and immunity of the host, and promote the translation of viral replication despite the activation of the interferon pathway [36,40,41]. Paradoxically and interestingly, although Arg seems to facilitate transcription of virus genes, its intestinal histological damage score, inflammatory factor level and oxidative stress index are significantly better than those of the PEDV group, which shows that the body can enhance its self-repair ability and anti-inflammatory response, and improve its disease tolerance [11,15,42,43]. These findings demonstrated that there was a complex interaction between host defense and virus reproduction. This dual effect necessitates further investigation into the potential impact of virus shedding and transmission, and balancing the benefits of host protection with the potential risk of increased virus load.

Inflammation is an important part of PEDV infection, which mainly leads to intestinal villi atrophy, crypt proliferation and intestinal mucosal barrier damage in weanling piglets [24,44]. The results showed that PEDV infection significantly up-regulated *IL-6*, *IL-8, IL-*1β and *CXCL2* in duodenum, ileum and colon. After oral administration of Arg, the expression of *IL-6* in ileum and *IL-6*, *CXCL2* and *REG3G* in colon were significantly decreased, but the expression of *IL-8* in ileum was increased. *IL-6* and *CXCL2* are intestinal pro-inflammatory factors, and Arg can significantly reduce their expression, which proves that Arg has anti-inflammatory effects and can reduce intestinal damage caused by PEDV infection [45,46,47]. Notably, Arg increased *IL-8* expression in the ileum, contrasting with its suppressive effects on *IL-6* and *CXCL2*. This may reflect compartmentalized immune regulation: *IL-8* is a potent neutrophil chemoattractant, and its up-regulation may represent a controlled recruitment of neutrophils to the ileum to facilitate localized pathogen clearance, whereas the suppression of *IL-6* prevents excessive inflammation. *REG3G* is an antimicrobial peptide involved in mucosal barrier and inflammatory response, and its overexpression may be associated with intestinal inflammation and intestinal mucosal damage caused by PEDV infection [48,49,50]. The results of this experiment showed that PEDV significantly up-regulated the expression of *REG3G*, but after the addition of Arg, *REG3G* decreased significantly in the colon, which provided effective support for proving that Arg had an anti-inflammatory function.

PEDV infection causes a strong antiviral response in the body, which is mainly manifested by the significant up-regulation of interferon-stimulated genes (ISGs) and interferon regulatory factors *(IRF7* and *IFN-β*). This indicates that the host mounts a robust innate antiviral response upon PEDV infection, in which type I interferon activates the JAK-STAT signaling pathway to stimulate the expression of ISGs and jointly resist viral replication and transmission [51,52,53]. In addition, the high expression of interferon-stimulating gene is accompanied by the transcriptional activation of IRF7, which is the key regulator to drive the type I interferon response in virus-infected intestinal epithelium [2,20,23]. The experimental results showed that the expression of antiviral genes (*MX1, OASL, ISG15* and *IFITM3*) in duodenum and ileum, and *MX1* and *OASL* in colon, were further enhanced after Arg administration [54]. This is consistent with previous studies; that is, Arg up-regulates *IFITM3, MX1* and *DHX58* in jejunum, indicating that Arg enhances the expression of interferon signal, which may be through the RIG-I-like receptor pathway. In addition, oral administration of Arg significantly up-regulated the expression of *IRF7* in duodenum and *IFN-β* in ileum, which indicated that Arg may act on the upstream of the IFN pathway and played an innate immune enhancement role by enhancing transcription amplification [55,56]. These findings suggest that Arg enhances innate antiviral responses. Synergistically augmenting this effect, Arg further fortified the innate immune ability by up-regulating interferon-stimulated genes, demonstrating its multifaceted role in host defense beyond mere antioxidant capacity.

Tissue repair after villi destruction caused by PEDV infection requires coordinated extracellular matrix remodeling and epithelial recovery. *MMP13* is a kind of collagenase, which degrades fibrocollagen and activates latent growth factor, which is very important for the update of extracellular matrix in the process of wound healing [57,58]. The experimental results showed that PEDV infection significantly increased the expression of *MMP13* in ileum, indicating that PEDV caused intestinal damage and intestinal repair processes were activated. After adding Arg, the expression of *MMP13* was further enhanced. The above results indicate that Arg can alleviate the intestinal damage caused by PEDV infection by promoting tissue repair and enhancing barrier integrity. Arg can facilitate viral transcription and protect the host at the same time, which distinguishes it from other nutritional interventions. N-Acetylcysteine (NAC), Puerarin (PR) and ellagic acid (EA) usually inhibit the replication of PEDV and restore the antioxidant capacity [20,59,60,61]. Tannic acid has been shown to enhance the body’s antioxidant capacity to alleviate the intestinal damage caused by PEDV infection [20]. In contrast, Arg increases the expression of virus genes and amplifies the host defense. This shows that Arg does not directly inhibit the virus, but enhances the host’s ability to tolerate and resist PEDV infection by reducing intestinal inflammation, enhancing the body’s antioxidant capacity and promoting intestinal tissue repair.

Distinct from conventional antiviral strategies that focus solely on pathogen clearance, our findings suggest that Arg promotes a state of disease tolerance, a novel perspective for nutritional intervention against viral infections [62]. This paradigm shift from solely targeting pathogen elimination to enhancing host resilience offers a promising avenue for developing complementary therapeutic strategies, particularly in contexts where complete viral eradication is challenging or undesirable. The follow-up research should focus on whether the combination of Arg and direct antiviral agents (such as NAC or EA) produces a synergistic protective effect, which is of great significance for its transformation into nutritional additives for preventing and treating PEDV.

## 5. Conclusions

In summary, while oral administration of 400 mg/kg BW Arg facilitates viral transcription to a certain extent, it significantly enhances the body’s antioxidant capacity, strengthens intestinal antiviral immunity, and regulates intestinal inflammatory responses. This unique dual action highlights Arg’s role in fostering disease tolerance, effectively alleviating intestinal damage caused by PEDV infection in piglets, and offering a novel perspective for nutritional intervention strategies. In this experiment, the tolerance of Arg to PEDV was studied, but whether it has the same effect on other coronaviruses is unknown, and further research is needed to explore the disease tolerance of Arg.

## Figures and Tables

**Table 1:** Effects of arginine supplementation on antioxidant status in PEDV-infected piglets.

ITEMS	−PEDV		+PEDV		SEM	*p*-Value		
	−Arg	+Arg	−Arg	+Arg		PEDV	Arg	PEDV×Arg
CAT								
Plasma	4.89 ^a^	4.09 ^b^	1.58 ^d^	2.93 ^c^	0.20	<0.001	0.182	<0.001
Duodenum	6.36 ^a^	5.29 ^a^	2.56 ^b^	2.71 ^b^	0.294	<0.001	0.128	0.047
Jejunum	9.78 ^a^	8.82 ^a^	6.20 ^b^	6.99 ^b^	0.406	<0.001	0.838	0.040
Ileum	1.38 ^b^	1.21 ^b^	1.34 ^b^	2.06 ^a^	0.113	0.001	0.022	0.001
Colon	3.68	3.97	3.85	4.87	0.465	0.260	0.169	0.443
T-SOD								
Plasma	91.46 ^a^	90.40 ^a^	80.88 ^b^	90.35 ^a^	1.272	<0.001	0.003	<0.001
Duodenum	212.06 ^b^	203.96 ^b^	209.71 ^b^	272.40 ^a^	7.121	<0.001	0.001	<0.001
Jejunum	359.14	349.49	302.68	273.69	7.309	<0.001	0.013	0.197
Ileum	209.75 ^a^	182.67 ^bc^	169.06 ^c^	186.58 ^b^	4.415	<0.001	0.288	<0.001
Colon	118.58	121.96	115.30	112.99	3.815	0.119	0.889	0.462
GSH-Px								
Plasma	530.82	480.02	463.94	514.57	18.036	0.531	0.997	0.057
Duodenum	59.75	61.29	61.88	61.22	2.371	0.759	0.897	0.745
Jejunum	59.58 ^b^	52.58 ^b^	63.70 ^b^	83.99 ^a^	3.688	0.002	0.213	0.014
Ileum	88.73 ^b^	82.63 ^b^	87.80 ^b^	126.86 ^a^	3.394	<0.001	0.002	<0.001
Colon	113.09	123.50	99.53	110.75	4.922	0.069	0.132	0.954
MDA								
Plasma	3.20	2.66	3.56	3.65	0.218	0.005	0.309	0.165
Duodenum	1.26	1.32	0.86	0.62	0.124	<0.001	0.463	0.237
Jejunum	0.57 ^ab^	0.57 ^ab^	0.69 ^a^	0.49 ^b^	0.047	0.666	0.034	0.038
Ileum	0.91	0.77	0.89	0.76	0.063	0.807	0.044	0.977
Colon	0.45	0.37	0.47	0.37	0.037	0.841	0.018	0.893
MPO								
Plasma	48.83	47.09	73.64	76.17	2.760	<0.001	0.892	0.448
Duodenum	0.48	0.46	0.54	0.47	0.035	0.362	0.223	0.550
Jejunum	0.40	0.43	0.48	0.49	0.032	0.040	0.494	0.799
Ileum	0.26	0.28	0.35	0.31	0.022	0.016	0.583	0.176
Colon	0.18 ^b^	0.20 ^b^	0.29 ^a^	0.21 ^ab^	0.023	0.011	0.211	0.047
H_2_O_2_								
Plasma	29.49	28.67	41.84	40.07	3.334	<0.001	0.700	0.888
Duodenum	8.03	7.89	9.52	9.32	0.598	0.021	0.780	0.964
Jejunum	14.38	13.82	12.59	10.68	0.332	<0.001	0.001	0.052
Ileum	2.56	1.90	3.07	2.22	0.328	0.215	0.029	0.766
Colon	11.17	11.73	10.37	10.19	0.673	0.094	0.780	0.586

Total superoxide dismutase (T-SOD); Catalase (CAT); Myeloperoxidase (MPO); Malondialdehyde (MDA); Glutathione peroxidase (GSH-Px); Hydrogen peroxide (H_2_O_2_). Values are mean and pooled SEM, *n* = 8. ^a,b,c,d^ Within a row, means with different superscripts differ (*p* < 0.05).

**Table 2:** Effect of arginine supplementation on complete blood count in piglets infected with porcine epidemic diarrhea virus.

ITEMS	−PEDV		+PEDV		SEM	*p*-Value		
	−Arg	+Arg	−Arg	+Arg		PEDV	Arg	PEDV×Arg
WBC (10^^9^/L)	13.01	14.19	14.12	12.98	1.460	0.975	0.992	0.432
Neu (10^^9^/L)	1.12	1.46	1.53	1.74	0.477	0.473	0.577	0.896
Lym (10^^9^/L)	11.60	12.45	12.32	10.97	1.461	0.798	0.863	0.458
Mon (10^^9^/L)	0.14	0.10	0.16	0.17	0.040	0.244	0.687	0.620
Eos (10^^9^/L)	0.14	0.18	0.10	0.09	0.030	0.038	0.534	0.481
PLT (10^^9^/L)	524.13	461.13	390.63	428.38	34.291	0.098	0.797	0.308
Neu (%)	9.19	11.75	11.08	12.50	2.644	0.727	0.598	0.880
Lym (%)	88.73	86.33	87.06	85.53	2.852	0.762	0.629	0.916
Mon (%)	1.01	0.64	1.14	1.19	0.179	0.194	0.527	0.409
Eos (%)	1.05	0.91	1.05	1.29	0.158	0.067	0.507	0.698
HCT (%)	37.36	37.50	38.42	32.50	7.725	0.425	0.247	0.225
RDW-CV (%)	28.29	29.31	27.89	29.31	0.634	0.183	0.825	0.051
PCT (%)	0.47	0.42	0.37	0.40	0.030	0.149	0.916	0.369
RBC (10^^12^/L)	5.19	5.00	5.27	5.30	0.105	0.199	0.599	0.449
HGB (g/L)	113.88	113.00	115.00	116.00	1.597	0.328	0.913	0.622
MCV (fL)	72.20 ^ab^	75.29 ^a^	73.03 ^ab^	70.03 ^b^	0.864	0.080	0.972	0.019
MCH (pg)	22.03	22.71	21.84	22.00	0.287	0.276	0.303	0.522
MCHC (g/L)	305.25 ^ab^	301.63 ^ab^	299.25 ^b^	313.88 ^a^	2.282	0.341	0.099	0.009
RDW-SD (fL)	70.81 ^ab^	79.16 ^a^	73.06 ^ab^	65.89 ^b^	2.007	0.062	0.838	0.011
MPV (fL)	8.98	9.26	9.39	9.44	0.161	0.207	0.464	0.606
PDW	15.68	15.79	15.59	15.6	0.090	0.409	0.465	0.883

Note: Values are mean and pooled SEM, *n* = 8. ^a,b^ Within a row, means with different superscripts differ (*p* < 0.05). White Blood Cell count (WBC), Neutrophil count (Neu), Lymphocyte count (Lym), Monocyte count (Mon), Eosinophil count (Eos), Platelet count (PLT), Hematocrit (HCT), Red cell Distribution Width—Coefficient of Variation (RDW-CV), Platelet Crit (PCT), Red Blood Cell count (RBC), Hemoglobin (HGB), Mean Corpuscular Volume (MCV), Mean Corpuscular Hemoglobin (MCH), Mean Corpuscular Hemoglobin Concentration (MCHC), Red cell Distribution Width—Standard Deviation (RDW-SD), Mean Platelet Volume (MPV), Platelet Distribution Width (PDW).

**Table 3:** Effect of arginine addition on PEDV structural genes.

ITEMS	−PEDV		+PEDV		SEM	*p*-Value		
	−Arg	+Arg	−Arg	+Arg		PEDV	Arg	PEDV×Arg
Duodenum	*/*	*/*						
*PEDV M*	*/*	*/*	1.000	5.338	0.567	<0.001		
*PEDV N*	*/*	*/*	1.000	1.918	0.156	<0.001		
*PEDV S*	*/*	*/*	1.000	2.198	0.181	<0.001		
Ileum								
*PEDV M*	*/*	*/*	1.000	3.377	0.339	<0.001		
*PEDV N*	*/*	*/*	1.000	2.484	0.102	<0.001		
*PEDV S*	*/*	*/*	1.000	2.519	0.228	<0.001		
Colon								
*PEDV M*	*/*	*/*	1.000	2.189	0.182	<0.001		
*PEDV N*	*/*	*/*	1.000	1.681	0.126	0.003		
*PEDV S*	*/*	*/*	1.000	1.623	0.122	0.006		

Note: PEDV M, Porcine Epidemic Diarrhea Virus Membrane protein; PEDV N, Porcine Epidemic Diarrhea Virus Nucleocapsid protein; PEDV S, Porcine Epidemic Diarrhea Virus Spike protein.

**Table 4:** Effect of arginine addition on genes related to antioxidant and tissue repair.

ITEMS	−PEDV		+PEDV		SEM	*p*-Value		
	−Arg	+Arg	−Arg	+Arg		PEDV	Arg	PEDV×Arg
Duodenum	/	/						
MX1	1.000 ^c^	0.786 ^c^	2.956 ^b^	5.723 ^a^	0.385	<0.001	<0.001	<0.001
OASL	1.000 ^c^	0.294 ^c^	4.510 ^b^	6.843 ^a^	0.511	<0.001	0.044	<0.001
ISG15	1.000 ^c^	0.285 ^d^	1.878 ^b^	3.721 ^a^	0.234	<0.001	<0.001	<0.001
IFITM3	1.000 ^c^	0.694 ^c^	3.135 ^b^	5.770 ^a^	0.394	<0.001	0.001	<0.001
IRF7	1.000 ^c^	0.704 ^c^	1.651 ^b^	2.112 ^a^	0.121	<0.001	0.527	0.007
AREG	1.000	1.257	5.955	5.955	0.476	<0.001	0.758	0.758
MMP13	1.000	1.680	1.786	2.249	0.114	<0.001	0.002	0.530
Ileum								
IFN-β	/	/	1.000	5.176	0.579	<0.001		
OASL	1.000 ^c^	0.996 ^c^	5.611 ^b^	8.848 ^a^	0.630	<0.001	0.004	<0.001
ISG15	1.000 ^c^	0.557 ^c^	3.383 ^b^	4.175 ^a^	0.292	<0.001	0.454	<0.001
MX1	1.000 ^c^	0.928 ^c^	2.968 ^b^	4.538 ^a^	0.301	<0.001	0.025	<0.001
IFITM3	1.000 ^c^	0.567 ^c^	3.169 ^b^	5.567 ^a^	0.387	<0.001	0.022	<0.001
IRF7	1.000	0.761	1.686	1.484	0.086	<0.001	0.067	0.871
AREG	1.000	1.729	26.391	25.580	2.396	<0.001	0.983	0.694
MMP13	1.000 ^c^	1.583 ^c^	8.411 ^b^	16.571 ^a^	1.221	<0.001	<0.001	<0.001
Colon								
OASL	1.000 ^a^	1.016 ^a^	0.298 ^c^	0.660 ^b^	0.064	<0.001	0.022	0.034
IFITM3	1.000 ^a^	0.656 ^b^	0.656 ^b^	0.795 ^b^	0.042	0.159	0.156	0.002
ISG15	1.000	0.651	0.490	0.231	0.054	<0.001	<0.001	0.269
MX1	1.000 ^bc^	1.236 ^b^	0.780 ^c^	2.337 ^a^	0.120	<0.001	<0.001	<0.001
IRF7	1.000 ^b^	0.971 ^b^	0.923 ^b^	1.719 ^a^	0.077	0.003	0.001	<0.001
AREG	1.000	0.917	2.515	2.904	0.184	<0.001	0.432	0.230
MMP13	1.000	1.399	2.099	2.596	0.138	<0.001	0.015	0.780

Note: OASL, 2′-5′-Oligoadenylate Synthetase-Like; ISG15, Interferon-Stimulated Gene 15; MX1, MX Dynamin-Like GTPase 1; IFITM3, Interferon-Induced Transmembrane protein 3; IRF7, Interferon Regulatory Factor 7; AREG, Amphiregulin; MMP13, Matrix Metallopeptidase 13; IFN-β, Interferon beta. *n* = 8. ^a,b,c,d^ Within a row, means with different superscripts differ (*p* < 0.05).

**Table 5:** Effect of arginine addition on genes related to intestinal inflammation.

ITEMS	−PEDV		+PEDV		SEM	*p*-Value		
	−Arg	+Arg	−Arg	+Arg		PEDV	Arg	PEDV×Arg
Duodenum	/	/						
*REG3G*	1.000	2.401	103.191	133.094	9.970	<0.001	0.060	0.085
*IL-1β*	1.000	1.110	13.692	16.839	1.369	<0.001	0.098	0.122
*IL-6*	1.000	1.191	2.519	2.426	0.143	<0.001	0.747	0.353
*CXCL2*	1.000	1.207	12.039	11.446	0.892	<0.001	0.801	0.604
*IL-8*	1.000	1.257	3.038	2.684	0.163	<0.001	0.797	0.114
Ileum								
*REG3G*	1.000	0.701	69.881	61.364	6.011	<0.001	0.150	0.179
*IL-6*	1.000 ^ab^	1.122 ^a^	0.871 ^bc^	0.733 ^c^	0.040	0.001	0.908	<0.001
*IL-1β*	1.000 ^c^	1.060 ^c^	3.051 ^b^	3.889 ^a^	0.251	<0.001	0.072	<0.001
*IL-8*	1.000 ^c^	1.400 ^c^	3.102 ^b^	4.597 ^a^	0.285	<0.001	0.001	0.042
*CXCL2*	1.000	1.393	2.473	2.811	0.157	<0.001	0.043	0.873
Colon								
*REG3G*	1.000 ^c^	3.698 ^b^	24.828 ^a^	2.652 ^bc^	1.774	<0.001	<0.001	<0.001
*IL-6*	1.000 ^bc^	0.690 ^c^	2.146 ^a^	1.310 ^b^	0.114	<0.001	<0.001	0.044
*IL-1β*	1.000	0.694	3.498	2.515	0.231	<0.001	0.008	0.147
*CXCL2*	1.000 ^c^	1.042 ^c^	4.400 ^a^	1.825 ^b^	0.257	<0.001	<0.001	<0.001
*IL-8*	1.000	1.407	2.283	2.291	0.128	<0.001	0.220	0.237

Note: IL-6, Interleukin 6; IL-8, Interleukin 8; CXCL2, C-X-C Motif Chemokine Ligand 2; REG3G, Regenerating Family Member 3 Gamma. *n* = 8. ^a,b,c^ Within a row, means with different superscripts differ (*p* < 0.05).

## Data Availability

Dataset available on request from the authors.

## Abbreviations

The following abbreviations are used in this manuscript:

PEDV	Porcine Epidemic Diarrhea Virus
Arg	*L*-Arginine
NO	Nitric oxide
LPS	Lipopolysaccharide
WBC	White Blood Cell Count
Neu	Neutrophil Count
Lym	Lymphocyte Count
Eos	Eosinophil Count
PLT	Platelet Count
RDW-SD	Red Cell Distribution Width—Standard Deviation
RDW-CV	Red Cell Distribution Width—Coefficient of Variation
MPV	Mean Platelet Volume
RBC	Red Blood Cell Count
HGB	Hemoglobin
MCHC	Mean Corpuscular Hemoglobin Concentration
MCH	Mean Corpuscular Hemoglobin
PDW	Platelet Distribution Width
PCT	Platelet Crit
MCV	Mean Corpuscular Volume
HCT	Hematocrit
CAT	Catalase
H_2_O_2_	Hydrogen Peroxide
T-SOD	Total Superoxide Dismutase
MDA	Malondialdehyde
MPO	Myeloperoxidase
GSH-Px	Glutathione Peroxidase
*PEDV M*	Porcine Epidemic Diarrhea Virus Membrane Protein
*PEDV N*	Porcine Epidemic Diarrhea Virus Nucleocapsid Protein
*PEDV S*	Porcine Epidemic Diarrhea Virus Spike Protein
*OASL*	2′-5′-Oligoadenylate Synthetase-Like
*ISG15*	Interferon-Stimulated Gene 15
*IL-6*	Interleukin 6
*MX1*	MX Dynamin-Like GTPase 1
*AREG*	Amphiregulin
*MMP13*	Matrix Metallopeptidase 13
*IFN-**β*	Interferon Beta
*IRF-7*	Interferon Regulatory Factor 7
*IFITM3*	Interferon-Induced Transmembrane Protein 3
*IL-8*	Interleukin 8
*CXCL2*	C-X-C Motif Chemokine Ligand 2
*REG3G*	Regenerating Family Member 3 Gamma
*IL-1**β*	Interleukin 1 Beta
*RPL19*	Ribosomal Protein L19
NAC	N-Acetylcysteine
PR	Puerarin
EA	Ellagic Acid

## Appendix A

**Table A1:** Composition and nutrient contents of diet (whole milk powder) in present study.

Items	Energy (kJ)	Crude Protein (g)	Crude Fat (g)	Carbohydrate (g)	Na (mg)	Ca (mg)
Nutrients (per 100 g)	2131	24.20	28.60	38.90	264	875
Nutrients (%)	25	40	48	13	13	109

**Table A2:** Sequences of the primers used for quantitative RT-PCR analysis.

GENE	NCBI Accession Number	Primer Sequence (5′→3′)
PEDV M	NC_003436.1	F: TCCCGTTGATGAGGTGAT
		R: AGGATGCTGAAAGCGAAAA
PEDV N		F: TTGGTGGTAATGTGGCTGTTC
		R: TGGTTTCACGCTTGTTCTTCTT
PEDV S		F: CTCTCTGGTACAGGCAGCAC
		R: GCTCACGTAGAGTCAAGGCA
OASL	NM_001031790.1	F: GGCACCCCTGTTTTCCTCT
		R: AGCACCGCTTTTGGATGG
ISG15	NM_001128469.3	F: AGCATGGTCCTGTTGATGGTG
		R: CAGAAATGGTCAGCTTGCACG
IL-6	NM_214399.1	F: TACTGGCAGAAAACAACCTG
		R: GTACTAATCTGCACAGCCTC
MX1	NM_214061.2	F: AGTGCGGCTGTTTACCAAG
		R: TTCACAAACCCTGGCAACTC
AREG	NM_214376.1	F: GAGTACGATAACGAACCGCACA
		R: TTTCCACTTTTGCCTCCCTTT
MMP13	XM_021062710.1	F: AGTTTGGCCATTCCTTAGGTCTTG
		R: GGCTTTTGCCAGTGTAGGTATAGAT
IFN-β	NM_001003923.1	F: AGCAGATCTTCGGCATTCTC
		R: GTCATCCATCTGCCCATCAA
IRF7	NM_001097428.1	F: CAGAAGCAGCTCCACTACAC
		R: CTCCCAGTAGACTTTGCACTT
IFITM3	NM_001201382.2	F: CAACATCCGAAGCGAGACC
		R: AGTGGTGCAAACGATGATGAA
IL-8	NM_213867.1	F: TTCGATGCCAGTGCATAAATA
		R: CTGTACAACCTTCTGCACCCA
CXCL2	NM_001001861.2	F: CGGAAGTCATAGCCACTCTCAA
		R: CAGTAGCCAGTAAGTTTCCTCCATC
REG3G	NM_001144847.1	F: GAAGATTCCCCAGCAGACAC
		R: AGGACACGAAGGATGCCTC
IL-1β	NM_001302388.2	F: CAACGTGCAGTCTATGGAGT
		R: GAGGTGCTGATGTACCAGTTG
RPL19	NC_010454.4	F: AACTCCCGTCAGCAGATCC
		R: AGTACCCTTCCGCTTACCG
